# Avoiding a lost generation of scientists

**DOI:** 10.7554/eLife.17393

**Published:** 2016-05-13

**Authors:** Justin Q Taylor, Peter Kovacik, James Traer, Philip Zakahi, Christine Oslowski, Alik S Widge, Christin A Glorioso

**Affiliations:** 1 Massachusetts Institute of Technology, Cambridge, United States; 2 Department of Chemical Engineering, Massachusetts Institute of Technology, Cambridge, United States; 3 Department of Brain and Cognitive Sciences, Massachusetts Institute of Technology, Cambridge, United States; 4 Sloan School of Management, Massachusetts Institute of Technology, Cambridge, United States; 5 Department of Biological Sciences, Bridgewater State University, Bridgewater, United States; 6 Department of Psychiatry, Massachusetts General Hospital and Harvard Medical School, Boston, United States; 7 Picower Institute for Learning & Memory, Massachusetts Institute of Technology, Cambridge, United States; 8 Department of Biology, Massachusetts Institute of Technology, Cambridge, United States

**Keywords:** science policy, postdoc, careers in science, funding, early career researcher, advocacy

## Abstract

By sharing their experiences, early-career scientists can help to make the case for increased government funding for researchers.

Funding for academic research in the United States has declined to a 40-year low in real terms, and other countries are experiencing similar declines. This persistent shortage of support threatens to create a "lost generation" of researchers – talented scientists who either leave the profession entirely, or who stay but acquire the cynicism and short-term thinking that hinders progress. While all researchers are being affected by the decline in funding, early-career researchers such as postdoctoral fellows and new investigators are being hit hardest.

To address this issue, we created Academics for the Future of Science, an organization dedicated to giving a voice to early-career researchers. As part of our effort to engage and educate these researchers (Academics for the Future of Science, 2015) we have solicited stories, especially from postdocs and new investigators, about their experiences in the current funding climate. Each account that follows highlights a different but equally pernicious problem facing early-career researchers.

## Breeding cynicism and discouraging future generations: Dr. G

"I was a postdoc at [Ivy League university]. About half our lab depended on an NIH U-series grant [a large but milestone-driven research grant] that was up for renewal. Our PI assured us that even if that wasn't renewed, we could get other grants to cover the four postdocs. So, we worked hard, we met the milestones on the U, and we published the results in [a Nature specialty journal]. They still didn't renew us. The PI wrote five different grants that year. One was funded, but not in time. All four of us lost our jobs. I went back to Europe and the other three scrambled for industry positions. I am currently doing a second postdoc, but I still have my doubts about staying in science. Constant fighting over funding is not something I want to do for the next forty years, and things do not seem to be getting better".

Historically, mentors have taught their trainees that hard work that results in solid scientific findings will ultimately be rewarded. Dr. G's experience suggests that this no longer holds true, and this grim outlook was echoed throughout our interviews. Another postdoc told us how they felt the need to make undergraduates who are thinking of doing research aware of the situation: “I’ve had to have very frank discussions about the funding situation that we have now, and really make them aware that their dream job of being a professor may not be available to them.”

One faculty member we spoke with predicted that, as advisors have increasing trouble renewing grants, students who join a lab with grant support for a few years may go unpaid if the grant is not renewed. A few such cases could have a broad chilling effect on the willingness of students to pursue a PhD.

## Lost talent and missed opportunities: Dr. B

"The year I finished my PhD, my advisor won [a major national award], based partly on my work. They called it 'genius' and 'incredibly innovative.' I finished my PhD, spent a few months in the lab to wrap up, then took my first faculty job at a university across town, which is pretty normal for my field. They were excited to hire a female engineer. I felt proud of my work. But then, when I tried to take the next step, the funding dried up. NIH said it was too pre-clinical and the study section wasn't sure it would work. The US National Science Foundation (NSF) said it was too clinically oriented. Career award reviewers at both agencies felt there wasn’t enough evidence of university financial commitment, because I was technically in a soft money job. Nobody was willing to take a risk.

We struggled by, and I kept applying. I eventually landed about half a million [US Dollars] in grants over six years, but all that time I hated the panic of wondering if I would lose my job the next year for lack of funding. I was on track as far as promotion and tenure were concerned, but even so, I resigned after those six years, using the excuse that my family and I had decided to move to another state. We never went, and now I work full time for a technology startup. I miss the prestige of having a faculty position. But I don’t miss much else.”

Dr B.’s experience illustrates a disturbing consequence of the current funding climate – the fact that the average age at which a researchers gets their first grant is increasing. Roughly 3% of the recipients of R01 grants from the National Institutes of Health are 36 years old or younger, compared with 18% a few decades earlier (Rockey, 2012). As the prospects of a new professor securing a Federal grant decline, university departments must commit more internal funds to each new hire. The most entrepreneurial scientists, like Dr. B, may accept positions without guaranteed funding ("soft money"). Unfortunately, the limited resources of such jobs often place the investigator at a continued competitive disadvantage, limiting their success.

## The broad and hidden cost of instability: Dr. M

"I'm 10 years out from my PhD, and have become an expert in a cutting-edge subfield of neuroscience. I'm Instructor-level faculty at a top-tier medical center. One of my papers has over 500 citations. I've been asked to present at conferences around the world. So, when NIH put out a call for applications in my specialty, of course I applied.

I put in hours making sure I addressed all the requirements, but my proposal was rejected at the first review stage, before the formal meeting. Reviewers commented that I was too junior to be trying, regardless of my record or my co-investigators. It just feels like there’s a queue and I’m not at the front of it yet. I’m also applying for tenure track jobs. They’re telling me they receive 300 applications for each open position, but only really consider 5 or 10 applicants. If each of those who submit an application require three letters of reference, that’s 900 senior scientists who each take an hour of their time to write and submit a letter that won’t be read or used. This just wastes senior faculty's time.”

Dr. M followed a common strategy: compensating for low success rates by increasing the number of applications. However, every additional application takes time not only from the scientist who writes it, but the three (or more) who must then review it.

Research scientists must all become advocates, able to explain their work and career trajectories to non-scientists.

Early-career researchers like Drs. B and M have a reasonable response to uncertainty: taking actions to reduce it. Every hour spent managing this uncertainty is an hour spent away from actual science. The outcome is that early-career researchers are prevented from taking risks, innovating or prioritizing scientific discovery.

## Making early-career scientists' voices audible

We believe the appropriate policy solution is to increase government funding for all forms of scientific research. Further, we believe that a critical missing element from this conversation is stability – the importance of allowing good scientists to know that taking risks will not end their careers after decades of training and preparation. Today's powerhouse labs were built decades ago, in a funding climate in which it was possible for a brilliant early-career scientist to secure stable funding.

Science will not be given renewed priority unless legislators and policymakers hear from those on the front lines of the crisis. Research scientists must all become advocates, able to explain their work and career trajectories to non-scientists. Unfortunately, advocacy is not part of the graduate or postdoctoral curriculum.

**Figure 1.**
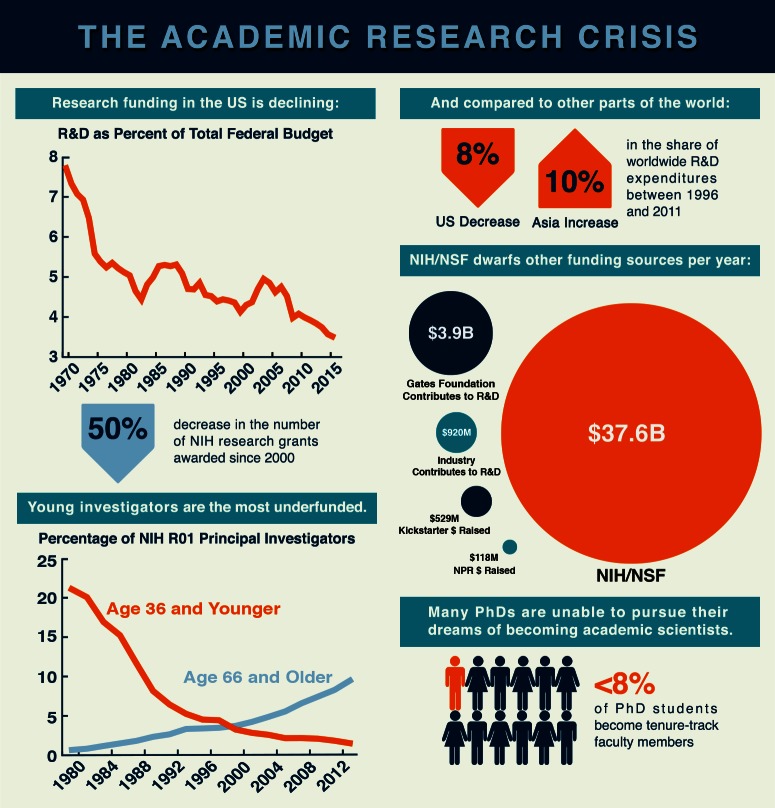
An example of advocacy. This infographic was prepared by Academics for the Future of Science to show how reduced levels of federal funding for scientific research adversely affect the career prospects of early-career researchers.

We propose two complementary strategies to bring advocacy organizations together with scientists for maximal effect:

**1. Building an informed and modern advocacy community**: A community of science advocates needs to be formed to achieve greater public funding. Communication is the key to collecting compelling stories from scientists and disseminating information about the state of science funding. As part of Academics for the Future of Science, we are working with local colleagues at MIT and the greater Boston area, as well as other organizations of scientists (such as Future of Research (www.futureofresearch.org), to modernize the way our community shares ideas about the uncertain future of science. Through our extensive online presence we have developed a number of resources for advocacy and we have collected many first-hand accounts of the effects of funding on scientists (Academics for the Future of Science, 2015). We continue to develop shareable information in the form of infographics (see Figure 1 for an example), short social media posts, and even cartoons. Social media and email allows us to reach out to early-career scientists, minorities and others underrepresented in the national dialogue. Social media in particular allows us to connect with scientists who express any level of interest (for example, by liking our Facebook page (www.facebook.com/academicsforthefutureofscience) or following us on Twitter (@SaveScience)), then gradually involve them further in advocacy through repeated follow-up.

**2. Community outreach:** Individual scientists have powerful stories to tell, but they are often too busy or removed from the political process to advocate for themselves. Advocacy organizations can help coordinate their efforts, and provide the tools and knowledge necessary for scientists to become self-advocates. Our organization has contributed to this goal by creating an online advocacy tool (http://save-science.org) to make it easier for busy scientists to communicate with their representatives. Open-source advocacy platforms now make it possible, in a few weeks, to deploy methods that were once available only to well-funded lobbying groups. We plan to introduce additional easy-to-use tools that enable scientists to represent themselves in legislative conversations. These are optimized for the US Congressional system, but could be tailored for a different representative body.

The three scientists who shared their stories above are examples of a much deeper problem, but they are also reason for hope. If more of these narratives can be placed in front of policymakers and the true cost of under-funding science made clear, the prospects for consistent funding for the next generation of scientists can improve. We hope that this article and the associated tools will be the beginning of a necessary change.

## References

[bib1] Academics for the Future of Science (2015). Academics for the Future of Science Facebook page and Website. https://www.facebook.com/academicsforthefutureofscience.

[bib2] Rockey S (2012). Age distribution of NIH principal investigators and medical school faculty. http://nexus.od.nih.gov/all/2012/02/13/age-distribution-of-nih-principal-investigators-and-medical-school-faculty/.

